# Graphene as a transparent conducting and surface field layer in planar Si solar cells

**DOI:** 10.1186/1556-276X-9-349

**Published:** 2014-07-13

**Authors:** Rakesh Kumar, Bodh R Mehta, Mehar Bhatnagar, Ravi S, Silika Mahapatra, Saji Salkalachen, Pratha Jhawar

**Affiliations:** 1 Thin Film Laboratory, Department of Physics, Indian Institute of Technology Delhi, Hauz Khas, New Delhi 110016, India; 2 Semiconductor Devices and Photovoltaics Department, Electronics Division, Bharat Heavy Electricals Limited, Mysore Road, Bangalore 560026, India

**Keywords:** Graphene, Solar cells, Front surface field, FDTD simulation

## Abstract

This work presents an experimental and finite difference time domain (FDTD) simulation-based study on the application of graphene as a transparent conducting layer on a planar and untextured crystalline *p*-*n* silicon solar cell. A high-quality monolayer graphene with 97% transparency and 350 Ω/□ sheet resistance grown by atmospheric pressure chemical vapor deposition method was transferred onto planar Si cells. An increase in efficiency from 5.38% to 7.85% was observed upon deposition of graphene onto Si cells, which further increases to 8.94% upon SiO_2_ deposition onto the graphene/Si structure. A large increase in photon conversion efficiency as a result of graphene deposition shows that the electronic interaction and the presence of an electric field at the graphene/Si interface together play an important role in this improvement and additionally lead to a reduction in series resistance due to the conducting nature of graphene.

## Background

Graphene has been considered as one of the promising materials for photovoltaic device applications due to its two-dimensional nature with extraordinary optical (transmittance ~98%), electronic (such as low resistivity, high mobility, and zero bandgap), and mechanical properties (Young's modulus 1.0 TPa) [1-3]. Many attempts have been made to utilize the extraordinary properties of graphene in electronic applications, such as solar cells, light-emitting diodes (LEDs), lithium-ion batteries, and supercapacitors. In particular, graphene can be used as an active (for electron-hole separation) or supporting layer in solar cell applications [4-11]. The superior flexibility and abundance of carbon source at lower costs make graphene a good alternative to indium tin oxide (ITO) as a transparent conducting electrode in numerous applications such as flexible solar cells, touch screens, and liquid crystal displays (LCDs) [12-14]. The advancements in the synthesis of large-area graphene with high crystallinity and transfer techniques make it suitable for its applications in solar cells [15].

In silicon solar cell, the power conversion efficiency is limited by many fundamental losses such as incomplete absorption of the solar spectrum, recombination of the photo-generated charge carriers, shading losses, and series resistance losses [16,17]. Antireflection coatings and passivation layers of oxides are used to overcome these losses [18,19]. Apart from these, front surface field (FSF) is also a very important technique to passivate the front surface by introducing an electric field at the surface to enhance the performance of silicon solar cell [20]. In a number of studies, the formation of a graphene/silicon (G/Si) junction for solar cell application has been studied. Li et al. reported the first demonstration on the G/Si solar cell with about 1.65% power conversion efficiency [21]. After that, many attempts have been made to improve the performance of graphene-based Si solar cells by modifying the work function and reducing the sheet resistance of graphene [22-25]. Although high optical transmittance and good electrical conductivity of graphene layer are well reported, there are limited studies in which the transparent conducting property has been studied by depositing the graphene layers onto fabricated solar cells. Difficulty in transferring a uniform graphene layer onto highly textured surfaces in normally available commercial-grade Si solar cells could be one of the possible reasons for this.

In this paper, we investigate the transparent conducting and surface field properties of graphene layers onto planar and untextured crystalline Si surface by carrying out experimental investigations and finite difference time domain (FDTD) calculations. In addition, the effect of graphene layer on the photovoltaic parameters and spectral responses of planar and untextured Si solar cell has also been investigated.

## Methods

### Synthesis and transfer of graphene

The growth of graphene has been carried out on a 25-μm-thick Cu foil (99.98%, Sigma-Aldrich, St. Louis, MO, USA, item no. 349208) using an atmospheric pressure chemical vapor deposition (APCVD) system at a temperature of 1,030°C. A split-type furnace with a quartz tube reactor was used for graphene growth. Before loading into the reaction tube, the Cu foil was cleaned in acetic acid followed by acetone, deionized water, and isopropyl alcohol to remove the copper oxide present at the surface. A mixture of Ar (500 sccm) and H_2_ (30 sccm) was then introduced into the reaction tube for degassing the air inside. The flow rate of Ar was kept constant (500 sccm) for all the experiments mentioned in this manuscript. The reactor was heated up to 1,030°C in 30 min, and this temperature was kept constant for the next 30 min to anneal the Cu foil. Then, CH_4_ (3 sccm) was fed into the reactor. After 30 min, the feeding of CH_4_ was cut off and the reactor was cooled down to room temperature naturally in an Ar and H_2_ environment. The flow of all the gases was stopped as the temperature reached close to the room temperature.

On successful growth of graphene on Cu foil, polymethyl methacrylate (PMMA) (Sigma-Aldrich, average *M*_W_ ~996,000, item no. 182265, 10 mg/ml in anisole) was used for the transfer of graphene onto different substrates like quartz, Si, SiO_2_-sputtered Si, and solar cells to study graphene quality and its electronic and optical properties. In the first step, the graphene-deposited Cu foil was attached to a glass slide with the help of a scotch tape and then PMMA was spin coated on one side of the Cu foil. The other side of the foil was immersed into 10% HNO_3_ solution for 2 min to etch out the graphene from that side. Subsequently, the Cu foil was etched using FeCl_3_ (10% wt./vol.) for 3–4 h. The PMMA coated graphene film was transferred to the desired substrate (quartz, Si or SiO_2_/Si, and solar cell) on several dips in deionized (DI) water as a cleaning step. In the final step, PMMA was etched out using acetone at 80°C for a duration of 2 h. Some residual PMMA was further removed by annealing in a H_2_ (500 sccm) and Ar (500 sccm) environment at a temperature of 450°C for 2 h.

### Solar cell fabrication

In order to study the effect of graphene on photon absorption and carrier collection, we first fabricated Si solar cells with planar and untextured surfaces. A 156-mm monocrystalline silicon wafer was dipped in high-concentration alkali solution at 80°C for 1 to 2 min to remove the roughness of the wafer. A *p*-*n* junction was then formed on the polished wafer through a high-temperature, solid-state diffusion process. Phosphorous oxy-chloride (POCl_3_) liquid dopant was used, and the wafers were subjected to elevated temperature in a furnace resulting in the formation of a thin layer of *n*-doped region (~0.5 μm). The wafers were etched using freon-oxygen (CF_4_) gas mixture in dry plasma etch machine to remove the junction regions created on the edge. These wafers were then chemically etched to remove the oxides and phosphorous glass formed on their surfaces. The entire backside was metallized with Ag-Al paste. Front contacts on the wafer surface were formed by screen printing the required pattern with a suitable metallic paste on them. The metal paste was dried and sintered in an infrared sintering belt furnace where temperature and belt speed were optimized to achieve a sharp temperature profile. The printed cells were then cut into smaller cells of dimension 10 mm × 10 mm for deposition of graphene. A similar printed cell is kept for comparative studies.

A 100-nm-thin film of SiO_2_ layer was deposited over mc-Si solar cell after the deposition of graphene layer using radio frequency (RF)-magnetron sputtering from a Si target (<111>) with 99.99% purity. The sputtering was carried out for 22 min by introducing Ar (15.8 sccm) and O_2_ (2.8 sccm) gases at room temperature with an applied RF power of 100 W.

### Characterization and measurements

Raman spectroscopic measurements were carried out in backscattering geometry using the 514.5-nm line of Ar^+^ laser for excitation. The scattered light was analyzed with a Renishaw spectrometer having a charged couple device for detection. All the optical measurements were carried out on a Lambda 35 UV/Vis spectrophotometer (PerkinElmer, Waltham, MA, USA). The photovoltaic characterization of the solar cell was carried out by measuring the *I*-*V* behavior using a 2400 SourceMeter (Keithley Instruments, Inc., Cleveland, OH, USA) under simulated AM 1.5 solar illumination at 100 mW/cm^2^ from a xenon arc lamp in ambient atmosphere.

## Results and discussion

**Figure 1:**
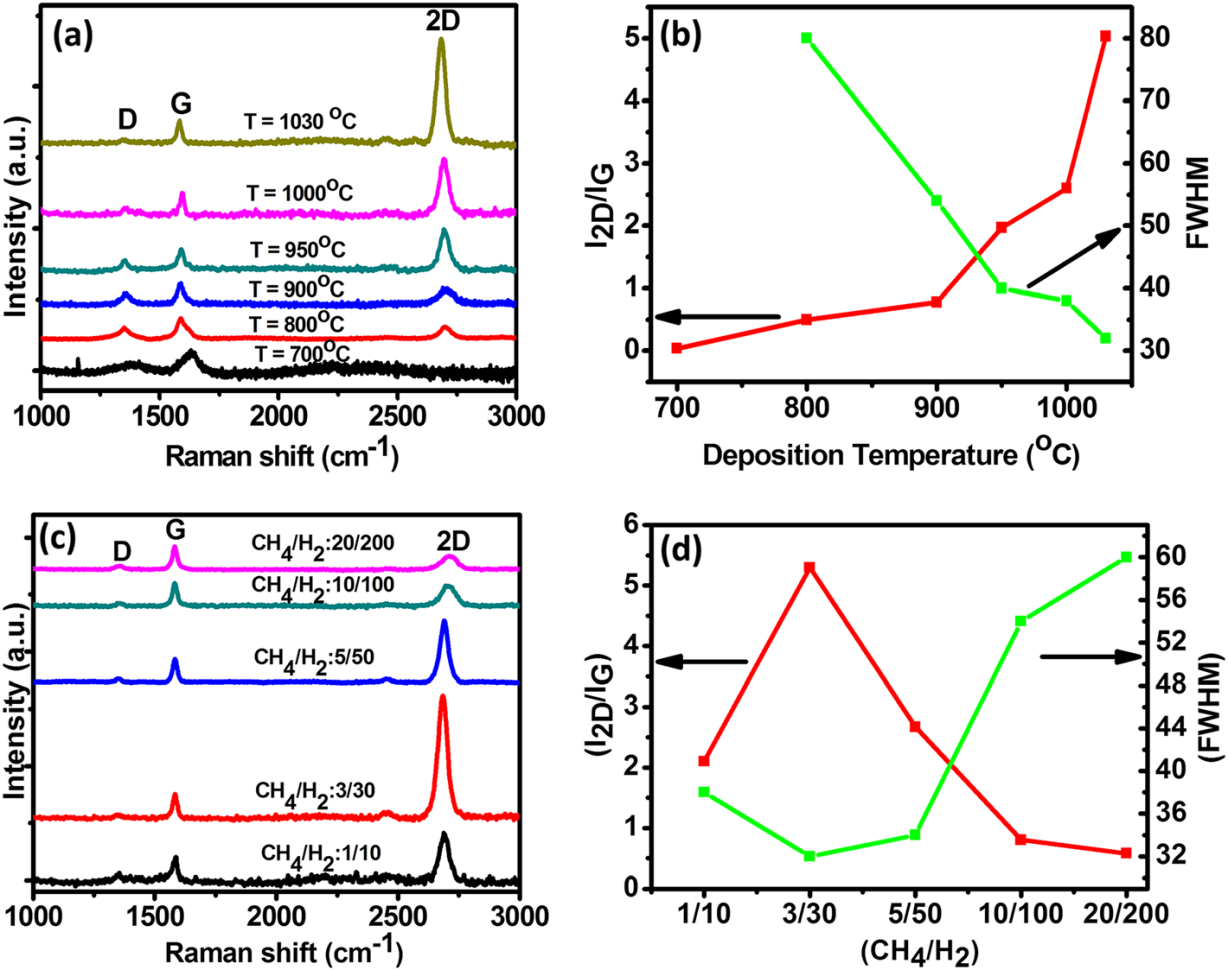
**Raman spectra and corresponding *I***_**2D**_**/*I***_**G **_**ratios of graphene at different temperatures and flow rates. (a)** Raman spectra of graphene synthesized at different growth temperatures and **(b)** corresponding *I*_2D_/*I*_G_ and FWHM of 2D peak. **(c)** Raman spectra of graphene synthesized with different flow rates of CH_4_ and H_2_ and **(d)** corresponding *I*_2D_/*I*_G_ and FWHM of 2D peak.

The APCVD conditions have been optimized to synthesize a single-layer graphene by tailoring the growth temperature and CH_4_/H_2_ flow rate. The quality of graphene was analyzed by Raman spectroscopy of the as-deposited graphene on the Cu foil. It is well known that graphene has three most prominent Raman features at ~1,350 cm^-1^ (D band), ~1,580 cm^-1^ (G band), and ~2,700 cm^-1^ (2D band). The D peak is related to the presence of defects (edges, dislocations, cracks, or vacancies) in graphene. The G peak denotes the symmetry-allowed graphite band corresponding to the in-plane vibration of *sp*^2^-hybridized carbon atoms, which constitute the graphene sheets. The 2D peak originates from the second-order double resonant Raman scattering from the zone boundary. It is quite established that Raman scattering can be used as a fingerprint for the quality and number of graphene layers. The ratio of the intensity of 2D and G peaks (*I*_2D_/*I*_G_) and full width at half maximum (FWHM) of the 2D peak are important parameters to evaluate the quality of graphene [26,27]. Figure 1a shows the Raman spectra of graphene films deposited on the Cu foil at different temperatures ranging from 700 to 1,030°C. At a temperature of 800°C or higher, the typical features of graphene, i.e., the 2D peak at 2,700 cm^-1^ and the G peak at 1,580 cm^-1^, are observed. It is worth noting that the defect-related D (near 1,350 cm^-1^) peak decreases with increase in temperature and finally disappears at a temperature of 1,030°C, indicating the improved quality of graphene deposited at higher temperatures. The improved quality of graphene is also confirmed by the *I*_2D_/*I*_G_ ratio and FWHM (2D) plots in Figure 1b, which show that the *I*_2D_/*I*_G_ ratio increases and FWHM (2D) decreases with increase in temperature.

In order to optimize the CH_4_/H_2_ flow rate for growing good-quality single-layer graphene, five flow rates of CH_4_/H_2_ content were chosen, i.e., 01/10, 03/30, 05/50, 10/100, and 20/200 sccm, while keeping the CH_4_:H_2_ flow rate ratio (1:10) constant. The growth temperature was set at the optimized value of 1,030°C with a deposition time of 30 min to ensure complete coverage of graphene. Raman spectra of graphene samples grown at different CH_4_/H_2_ flow rates are shown in Figure 1c, while the corresponding *I*_2D_/*I*_G_ ratio and FWHM data are shown in Figure 1d. The Raman spectra show very-low-intensity D peak (at ~1,353 cm^-1^) and large and symmetrical graphene G (~1,580 cm^-1^) and 2D (~2,700 cm^-1^) peaks. The D peak is negligible in all the cases, indicating a defect-free graphene growth. Furthermore, the FWHM of the 2D peak increases gradually from 30 to 65 cm^-2^ (as shown in Figure 1d) and the *I*_2D_/*I*_G_ peak ratio changes from 1.3 to 0.3. The optimal CH_4_/H_2_ ratio to produce monolayer graphene, determined experimentally, is 03/30. The decrease in *I*_2D_/*I*_G_ and increase in FWHM with the increase in CH_4_/H_2_ flow rate indicate an increase in the number of graphene layers upon increasing the CH_4_/H_2_ flow rate. The values of *I*_2D_/*I*_G_ (>5) and FWHM (≈32 cm^-1^) in graphene grown at 1,030°C and 03/30-sccm CH_4_/H_2_ flow rate match well with the previously reported values for monolayer graphene [26,28-30]. Based on the above study, graphene layer grown for 30 min at a deposition temperature of 1,030°C with 03 sccm of CH_4_ and 30 sccm of H_2_ flow rates was used for investigating the effect of graphene and G/SiO_2_ layers on Si solar cell as a transparent conducting and antireflection layer.

**Figure 2:**
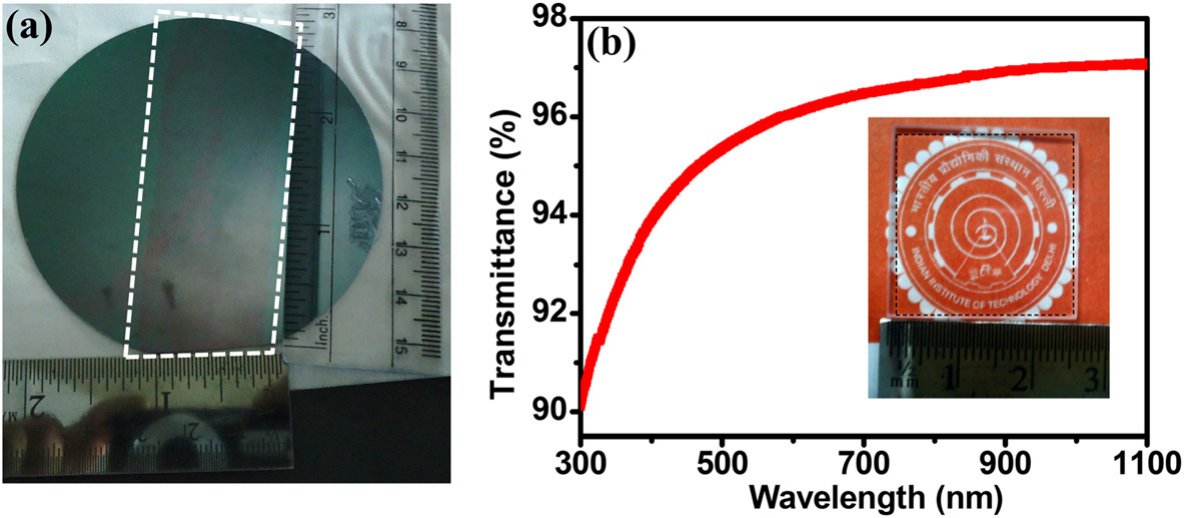
**Optical image and transmittance of graphene. (a)** Optical image of a large-area (~6.5 × 2.5 cm^2^) graphene transferred onto a SiO_2_ (300 nm)/Si substrate. **(b)** Transmittance of graphene after it was transferred onto a quartz substrate. The inset photograph of **(b)** shows the transparency of the transferred graphene sample.

Figure 2a shows the optical image of large-area (~6.5 × 2.5 cm^2^) graphene transferred onto a SiO_2_ (300 nm thick)/Si substrate. In order to measure the transmittance values, graphene layer was transferred to a quartz substrate and an average value of transmittance of 97% (Figure 2b) at a visible wavelength range of interest of 400 to 1,100 nm for Si solar cell was observed. A sheet resistance of graphene of about 350 Ω/□ was observed after transferring it on a SiO_2_ (300 nm)-coated Si substrate. A comparison of sheet resistance and transmittance of graphene layer used in studies involving G/Si cells is given in Table 1. As already mentioned, the central objective of the present study was to evaluate the potential advantages of using graphene as a transparent conducting and surface field layer for Si solar cell. A commercially available silicon solar cell has a Si_3_N_4_ antireflection layer along with a textured surface. It is difficult to deposit/transfer graphene layer on a textured surface. In order to study the transparent conducting properties of graphene layer, it is necessary to remove the Si_3_N_4_ layer and texturing of these cells. Therefore, the silicon solar cells with these properties, i.e., with planar Si surface, were fabricated for carrying out these experiments. The procedure followed for fabricating Si (*p*-*n*) solar cells with planar top surface (without texturing and AR coating) is described in the ‘Methods’ section. For applying graphene as a transparent conducting and surface field layer on Si solar cells, we chose SiO_2_ as the antireflection layer. Experimental and simulation studies were performed on the planar Si solar cell to investigate the reflectance properties of monolayer graphene on Si surface. Subsequently, the thickness of SiO_2_ layer as an antireflection coating for G/Si solar cell was optimized. It was observed that a 100-nm-thick SiO_2_ layer was sufficient to work as an antireflection layer over the graphene-Si interface. SiO_2_ (refractive index 1.45) was chosen due to its well-known antireflection properties [31].

**Table 1:** A comparison of transmittance and sheet resistance values of graphene layers used in reported studies on Si solar cells

	**Method of preparation**	**Transmittance (%)**	**Sheet resistance (Ω/□)**	**Efficiency (%)**
1	CVD using Cu foil	96 to 98	900	8.9 [24]
2	CVD using Cu foil	95 to 97	>1000	8.6 [23]
3	CVD using Ni foil	54 to 70	-	1.7 [21]
4	Fame synthesis using Ni foil	>75	-	4.3 [32]
5	CVD using Ni foil	-	200	2.8 [33]
6	CVD using Cu foil	97	350	8.94 (in the present study)

**Figure 3:**
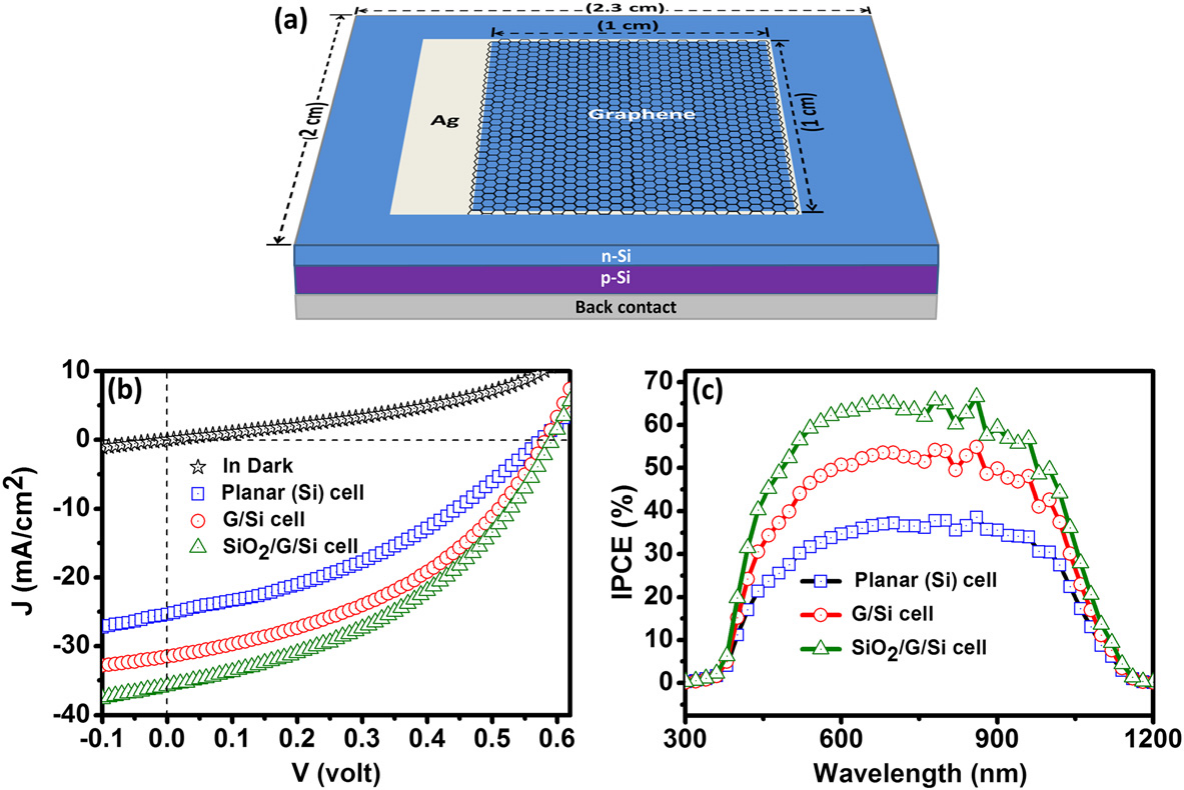
**Illustration, *J*-*V *characteristics, and IPCE of solar cells. (a)** The schematic diagram of the planar Si solar cell used in the present study showing Ag contacts, active area with graphene deposition, and different layers. **(b)** Dark and light *J*-*V* curves and **(c)** the IPCE of planar Si, G/Si, and SiO_2_/G/Si solar cells.

Graphene and SiO_2_/G overlayers with 100 nm SiO_2_ thickness were then applied onto the fabricated crystalline Si solar cell having a planar and untextured Si surface (Figure 3a) to experimentally determine the effect of these layers on the performance of solar cell. Figure 3b depicts the dark and illuminated *J*-*V* characteristics of (i) a bare Si solar cell having a planar surface, (ii) graphene on the planar Si solar cell (G/Si), and (iii) 100-nm-thick SiO_2_ coating on graphene/Si solar cell (SiO_2_/G/Si). The solar cell performance parameters of open circuit voltage (*V*_OC_), short circuit current density (*J*_SC_), maximum voltage (*V*_M_), maximum current (*I*_M_), series resistance (*R*_S_), shunt resistance (*R*_SH_), fill factor (FF), and the energy conversion efficiency (Eff.) are shown in Table 2. Data given in Table 2 shows an overall improvement in the performance of the planar Si solar cell with an increase in *V*_OC_ by 20 mV and in *J*_SC_ by 10.5 mA/cm^2^. It is important to note that the graphene overlayer on planar Si solar cell (G/Si) has higher conversion efficiency (7.85%) in comparison to the bare Si cell (5.38%) without graphene layer. This conversion efficiency is further increased to 8.94% on introduction of the antireflection SiO_2_ layer. Improvement in the *I*-*V* properties on deposition of graphene onto Si cells may be due to changes in the reflection properties of planar Si cells, improvements in the photovoltaic parameters due to transparent conducting properties of graphene, and the formation of surface electric field layer at the G/Si surface. To separate theses effects, reflectance and junction properties of the G/Si junctions were evaluated.

**Table 2:** **Performance parameters of planar (Si), G/Si, and SiO**_**2**_**/G/Si cells**

**Cell type**	***V***_**OC **_**(mV)**	***I***_**SC **_**(mA/cm**^**2**^**)**	***V***_**M **_**(mV)**	***I***_**M **_**(mA/cm**^**2**^**)**	***R***_**S **_**(Ω/cm**^**2**^**)**	***R***_**SH **_**(Ω/cm**^**2**^**)**	**FF (%)**	**IPCE (%) (at 600 nm)**	**Eff. (%)**
Planar (Si) cell	573.0	25.3	352.0	15.3	11.4	50.0	36.5	34.7	5.38
G/Si	582.0	31.5	383.0	20.5	6.2	70.0	42.5	50.5	7.85
SiO_2_/G/Si	593.0	35.8	387.0	23.1	5.8	53.2	42.6	62.7	8.94

**Figure 4:**
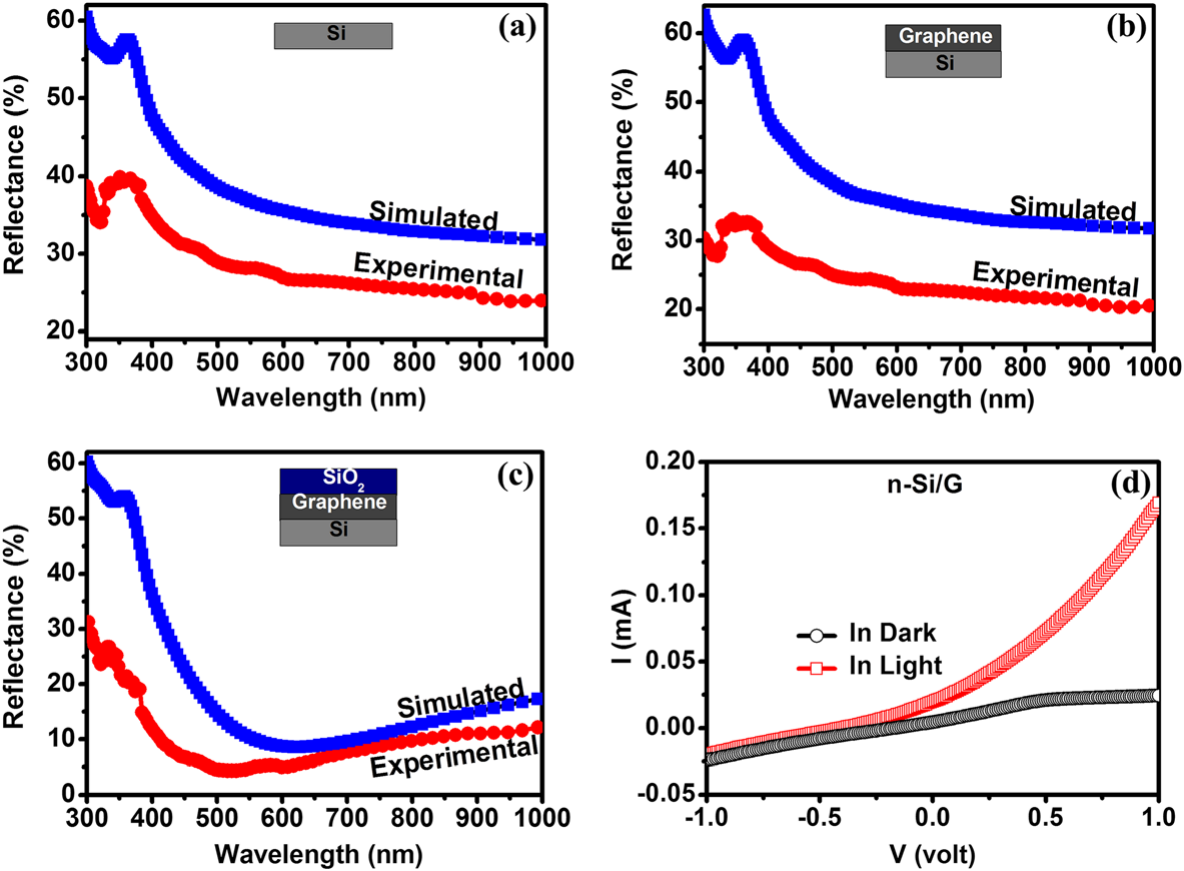
**Simulated and experimental reflectance spectra and current-voltage characteristics of solar cell samples.** Simulated and experimental reflectance of **(a)** Si cell, **(b)** G/Si cell, and **(c)** SiO_2_/G/Si cell. The thickness of SiO_2_ layer used was 100 nm. **(d)** Current-voltage characteristics for graphene/*n*-Si interface in dark and light.

**Figure 5:**
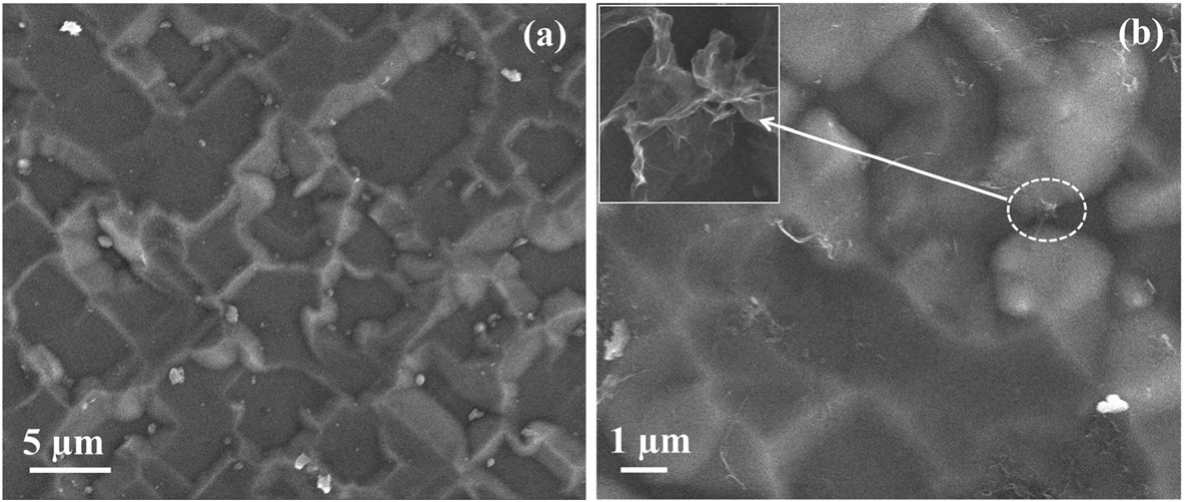
**FESEM images of planar Si solar cell surface.** FESEM image of the top surface of especially fabricated planar Si solar cell **(a)** before and **(b)** after transferring the graphene. Inset of **(b)** shows some wrinkles observed in the graphene on the planar Si surface.

Figure 4a shows the simulated and experimental reflectance spectra of polished Si and planar Si solar cell samples. The deviation of our simulated results from the experimental results may be attributed to the nature of Si surface in both cases. The FDTD simulations were carried out incorporating an ideal planar Si surface. The lower reflectance values in the experimentally measured reflectance spectra are attributed to some inherent roughness (Figure 5a) in the planar Si sample used for solar cell fabrication. In Figure 4b, the simulated and experimentally measured reflectance spectra of Si after deposition of monolayer graphene (G/Si) are plotted. It is clear from the simulated results (Figure 4a,b) that Si and G/Si samples do not show any difference in reflectance values. But, our experimental results (Figure 4a,b) show that the reflectance of Si reduces to about 4 to 5% on deposition of graphene on planar Si. Earlier, a reduction of about 70% in reflectance of Si has been reported to take place on deposition of graphene [21,34], although the thickness of graphene used was quite large (20 nm). Reductions of about 4 to 5% in the reflectance of planar Si on deposition of graphene in the wavelength range of interest are quite interesting. The difference in the simulated (Figure 4b) and experimental (Figure 4c) values is attributed to the deviation in the nature of ideal graphene layer used in simulation in comparison to that in the experiment. In the optical model for FDTD simulation, a wrinkle-free monolayer graphene deposited on the complete substrate area without the effect of the substrate is considered. However, it is well known that graphene obtained by any synthesis technique would have many defects in the form of wrinkles, ripples, ridges, folding, and cracks [35-37]. Additionally, some unwanted molecular doping such as water molecules may also be present on the surface of graphene [38,39]. These factors can modify its optical properties and thus the reflectance of G/Si structure [21,34,40]. Furthermore, it is also reported that the amount of wrinkles, folding, and chemical doping in graphene depends upon the substrate onto which graphene is deposited or transferred [39]. In an earlier study, it has been demonstrated that deposition of wrinkle-like graphene sheets exhibits a broadband light trapping effect in Al nanoparticles and graphene-based solar cells [41]. Thus, the observed decrease in reflectance in G/Si samples in comparison to the change in reflectance in the simulated results can be due to such adsorbed molecules or because of the synthesis defects and wrinkles (Figure 5b) in graphene.

The *I*-*V* behavior of graphene/Si (G/*n*-Si) structure was obtained to study the nature of G/*n*-Si junction. Figure 4d shows the *I*-*V* characteristics of the G/*n*-Si in dark and light. The forward bias condition was observed with graphene connected to the negative terminal with respect to *n*-Si. This shows that the interface between the graphene and *n*-Si behaves like a *n*^+^-*n* junction. The favorable direction of the electric field formed at the interface helps in the reduction of the effective recombination at the front surface and enhances the collection of light-generated free carriers and thus improves the efficiency of solar cell. The *n*-type or *p*-type nature of graphene is very sensitive to the synthesis method, adsorbed molecules, nature of the substrate underneath, etc. [42-45]. It can be conjectured that the graphene deposited onto Si (*n*-type) in G/Si cells in the present study acts like an *n*-type layer.

**Figure 6:**
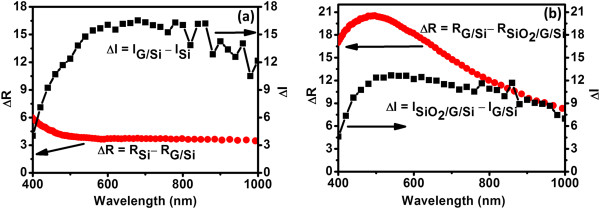
**Comparison of reflectance and IPCE of solar cells.** A decrease in the reflectance (∆*R*) and an increase in the IPCE (∆*I*) on going from Si to G/Si **(a)** and G/Si to SiO_2_/G/Si **(b)** solar cells.

A large increase in the short circuit current on graphene deposition onto planar Si solar cell is very interesting on various accounts. As mentioned earlier, there are two important contributions that might result in the enhancement in *J*_SC_ and conversion efficiency values as shown in Table 2. The first effect is due to the generation of surface field at the G/*n*-Si interface and reduction in the associated series resistance. The *J*-*V* curve (Figure 3b) shows a lower series resistance (*R*_S_) in G/Si cell (6.2 Ω) in comparison to pristine cell (11.4 Ω). It is important to note that the improvement in efficiency (2.47%) for Si solar cell by using graphene as a surface field layer is larger than or similar to the efficiency improvement (2.38%) obtained by using the *n*^+^ doping (thickness ≈ 2 × 10^20^ cm^-3^ and 0.07 μm) on the front surface [20]. The second effect is the decrease in reflectance of planar Si solar cell after graphene deposition. These improvements in *J*-*V* characteristics are further validated by the incident photon conversion efficiency (IPCE) measurements shown in Figure 3c. It is clear from the IPCE plot (Figure 3c) that both graphene and SiO_2_/G layers improve the photon to electron conversion ratio considerably compared to the bare planar Si solar cell. The decrease in the reflectance (∆*R*) of graphene-deposited Si (Figure 6a) is about 4 to 5% in the wavelength range of interest for Si solar cell. But, the increase in IPCE (∆*I*) is much larger than the decrease in reflectance (∆*R*) as one goes from Si to G/Si structure. This confirms that the electric field formed at the G/*n*-Si interface is aiding carrier collection. Thus, the deposition of graphene onto polished *n*-Si surface is aiding carrier collection or photon absorption in addition to lowering its reflectance. A slight increase in *V*_OC_ from 573 to 582 mV also indicates the active participation of graphene in the solar cell device. Earlier, a number of studies have reported the effect of graphene quality, number of graphene layers, and adsorbed molecules on the electronic properties of graphene-Si interface. Li et al. reported that the incorporation of graphene introduced a built-in electric field near the interface between the graphene and silicon (*n*-type) to help in the collection of photo-generated carriers [21]. Attention may also be paid to the study on the effect of the number of graphene layers and chemical doping on the properties of the graphene-Si interface [22,25,46]. Further, on deposition of SiO_2_ (on going from G/Si to SiO_2_/G/Si cell), the increase in IPCE is much smaller than the decrease in the reflectance value (Figure 6b). This clearly indicates that the main effect on SiO_2_ deposition is due to improvement in the antireflection properties only. The improvement in the *J*_SC_ on SiO_2_ deposition (on going from G/Si to SiO_2_/G/Si cell) is primarily due to the antireflection properties of the 100-nm-thick SiO_2_ layer. Consequently, the large improvement in *J*_SC_ and small increase in *V*_OC_ indicate that graphene behaves like an *n*^+^ layer which intrudes a surface field at the interface to enhance the collection of light-generated carriers thereby improving the efficiency of the *p*-*n* Si solar cell. Further, a decrease in the series resistance value and a small increase in *V*_OC_ on deposition of SiO_2_ layer on the G/Si cell are due to modification in the electronic properties of the G-Si interface during SiO_2_ deposition process. By modifying the electronic properties of graphene layer, the photovoltaic properties of silicon solar cell can be improved further.

## Conclusions

The present study is a clear demonstration of the useful combination of the properties of graphene: (i) as a transparent conducting layer, which provides high transmittance (97%) and reduces the series resistance of planar *p*-*n* Si solar cell; (ii) as an antireflection layer, which reduces the reflectance of the planar *p*-*n* Si solar cell due to the presence of wrinkles; and (iii) as a surface field layer onto *n*-type Si due to *n*^+^-*n* nature of the interface, which provides a favorable electric field for reducing the carrier recombination. Due to these effects, an increase in efficiency from 5.38% to 7.85% is observed. Deposition of a layer of SiO_2_ of an optimized thickness value leads to a further increase in the short circuit current density due to its antireflection properties.

## Competing interests

The authors declare that they have no competing interests.

## Authors' contributions

RK carried out all the experiments in this study, analyzed and interpreted the data, and drafted the manuscript. MB was involved in SiO_2_ deposition. SR, SM, SS, and PJ jointly fabricated the *p*-*n* Si solar cell. BRM supervised the overall study, analyzed the results, and finalized the manuscript. All authors read and approved the final manuscript.

## Authors' information

RK and MB are PhD students in the Department of Physics, IIT Delhi, India. BRM is a professor (Schlumberger Chair) in the Department of Physics, IIT Delhi, India. SM, SS, and PJ are photovoltaics engineers at BHEL, India.
